# Inhibition of Breast Cancer Cell Proliferation by 9-Hydroxycamptothecin-Loaded Zeolitic Imidazolate Nanoparticles

**DOI:** 10.32604/or.2025.066058

**Published:** 2025-09-26

**Authors:** Chuansheng Yang, Xiaoling Zhou, Ling Luo, Zirun Luo, Kaiming Fan, Chenglai Xia

**Affiliations:** 1 Department of Breast, Thyroid and Head-Neck Surgery, Yuebei People’s Hospital of Shantou University, Shaoguan, 512099, China; 2 School of Pharmaceutica Sciences, Southern Medical University, Guangzhou, 510515, China; 3 Scientific Research Center, Foshan Women and Children Hospital Affiliated with Guangdong Medical University, Foshan, 528000, China; 4 Department of Breast, Thyroid and Head-Neck Surgery, The Third Xiangya Hospital of Central Sounth University, Changsha, 410000, China; 5 Department of Anesthesiology, FangChengGang Hospital of Guangxi University of Chinese Medicine, FangChengGang, 538021, China

**Keywords:** 9-hydroxycamptothecin, nanometer, zeolimidazole, breast cancer, cell proliferation

## Abstract

**Objectives:**

Novel drug delivery systems have been designed to enhance local drug concentrations while reducing side effects conducive to improved breast cancer treatment outcomes. This study aimed to identify the anti-cancer function of zeolite imidazole ester-based material loaded with camptothecin nanoparticles.

**Methods:**

We utilized a zeolitic imidazolate backbone material to fabricate 9-hydroxycamptothecin nanoparticles and investigated their impact on breast cancer cell proliferation. Scanning electron microscopy and Fourier-transform infrared spectroscopy revealed changes in the carrier skeleton of the loaded 9-hydroxyl camptothecin, characterized by a reduction in surface smoothness, accompanied by slight collapses and folds on the particle surface. Notably, we detected vibration of the benzene ring in the 9-hydroxycamptothecin structure within the nanoparticles. Cell proliferation was tested by CCK-8. Protein expression was measured by Western blot. The efficacy of nanoparticles was evaluated by animal experiments.

**Results:**

In this study, we utilized a zeolitic imidazolate backbone material to fabricate 9-hydroxycamptothecin (9-HCPT) nanoparticles and investigated their impact on breast cancer cell proliferation. Scanning electron microscopy and Fourier-transform infrared spectroscopy revealed changes in the carrier skeleton of the loaded 9-hydroxyl camptothecin, characterized by a reduction in surface smoothness, accompanied by slight collapses and folds on the particle surface. Notably, we detected vibration of the benzene ring in the 9-HCPT structure within the nanoparticles. Using the CCK-8 method, we evaluated the inhibitory effect of these nanoparticles on breast cancer cells and observed a significant reduction in the cytotoxicity of camptothecin (CPT) when incorporated into the zeolite imidazole ester skeleton material. Immunoblot analysis showed upregulation of cyclic GMP-AMP synthase (cGAS), stimulator of interferon genes (STING), and NF-κB-p65 in response to the nanoparticles. These results showed that our nanoparticles might be a useful drug delivery strategy to overcome breast cancer drug resistance.

**Conclusion:**

The findings of this study suggest that nanoparticles loaded with CPT and formed from zeolite imidazole ester backbone material possess immune-enhancing properties that could suppress breast cancer progression. Accordingly, these nanoparticles hold promise as potential lead compounds for combined immunotherapy in breast cancer treatment.

## 1 Introduction

Breast cancer is a prevalent malignant tumor affecting women, with its incidence steadily increasing in recent years, posing a significant threat to women’s lives and health [1]. Current treatment methods for breast cancer include surgery, endocrine therapy, radiotherapy, and chemotherapy [2]. However, tumors often develop drug resistance, compromising the effectiveness of therapy [3]. It is now understood that tumor resistance is complex, involving key factors, notably inadequate local drug concentration reaching the tumor site and the creation of a tumor immunosuppressive microenvironment [4].

Camptothecin (CPT) is a class of pyrroquinoline alkaloids that reportedly targets topoisomerase I (TOP1) [5]. Its mechanism of action involves blocking DNA synthesis in the S phase of tumor cell replication by inhibiting topoisomerase I activity, leading to tumor cell apoptosis [6]. However, CPT’s clinical application is limited due to serious *in vivo* side effects, poor water solubility, rapid *in vivo* metabolism, low bioavailability, intravenous toxic side effects, and inadequate targeting.

Nano drugs, leveraging nanotechnology in drug delivery systems, offer significant advantages in the medical field [7]. They can refine drug water solubility, enhance drug metabolism kinetics, and improve safety and targeting abilities *in vivo* [8]. Nano drugs can be categorized into passive targeted nanoparticles, active targeted nanoparticles, and stimulus-responsive nanoparticles [9,10]. This study plans to identify the anti-cancer function of zeolite imidazole ester-based material loaded with camptothecin nanoparticles, and evaluate its function in cancer immunotherapy.

## 2 Materials and Methods

### 2.1 Reagents, Antibodies, and Cells

Human breast cancer cell lines MDA-MB231 cells, BT20 cells, and mouse breast cancer cell line 4T1 cells were bought from Shanghai ZBIO company (Shanghai, China) and cultured in a 37°C, 5% CO_2_ incubator using DMEM with RPMI-1640 medium supplemented with 10% fetal bovine serum (FBS), 100 U/mL streptomycin, and 100 U/mL penicillin. All cell lines used in this study have been authenticated (STR) and tested for mycoplasma contamination. The DMEM and RPMI-1640 media were purchased from Gibco (Thermo Fisher Scientific, Grand Island, NY, USA), while 9-HCPT was acquired from MCE (Mount Arlington, NJ, USA). CCK-8 reagent was obtained from Dojindo Laboratories (Kumamoto, Japan), and the rabbit anti-human monoclonal antibodies for cGAS (#79978, 1:1000 dilution), STING (#13647, 1:1000 dilution), NF-κB (#8242, 1:1000 dilution), GAPDH (#8884, 1:1000 dilution) and tubulin (#2144, 1:1000 dilution) were purchased from CST (Danvers, MA, USA). The HRP-labeled anti-rabbit secondary antibody (#7074) was also from CST (Danvers, MA, USA). For the synthesis and experiments, zinc nitrate hexahydrate (Zn (NO_3_)_2_·6H_2_O), 2-methimazole (2-MiM), dopamine (DA), folic acid (FA), fluorescein isothiocyanate (FITC), methanol (CH_3_OH), and ethanol (CH_3_CH_2_OH) were purchased from Shanghai Aladin Biochemical Technology Company (Shanghai, China). Trypsin was obtained from Hangzhou Nordend Biotechnology Company (Hangzhou, China), and 4^′^,6-diamidine-2-phenylsyl (DAPI) was purchased from Shanghai Beyotime Biotechnology (Shanghai, China).

### 2.2 Preparation of the Zeolite Imidazole Ester Backbone Material (ZIF-8)

First, 657 mg of 2-MiM and 297.5 mg of zinc nitrate hexahydrate were accurately weighed and dissolved in 10 mL of methanol, creating a homogeneously dispersed solution. The solution containing 2-Mimidazole was added to the zinc nitrate solution at room temperature, and the mixture was stirred by a magnetic stirrer (Biofuge Stratos, Thermo Fisher Scientific, Waltham, MA, USA) at a rate of 1200 rpm for 30 min. Following this, a white precipitate formed and was collected through centrifugation. The precipitate was washed thrice with deionized water to eliminate excess reactants. Finally, the white precipitate was collected and subjected to freeze-drying, resulting in the obtainment of ZIF-8 nanoparticles.

### 2.3 Preparation of the HCPT@ZIF-8

To produce ZIF-8 nanoparticles loaded with HCPT (HCPT@ZIF-8), 10 mg of ZIF-8 nanoparticles were dispersed in 10 mL of HCPT methanol solution with a 1 mg/mL concentration. The mixture was stirred for 24 h and then collected by centrifugation (1000 g/rcf) to obtain HCPT@ZIF-8. The concentration of unloaded HCPT in the centrifugal supernatant was determined by measuring the absorbance of HCPT at 390 nm using a UV spectrophotometer (UV-2700, Shimazu, Japan). The encapsulation efficiency (EE) and drug loading content (DLC) were calculated using the following equations.

$$\text{EE}(\%)=\frac{\text{Drug loading content}}{\text{Drug feeding content}}\times 100$$

$$\text{DLC}(\%)=\frac{\text{Mass of HCPT in complexes}}{\text{Total mass of complexes}}\times 100$$

### 2.4 Preparation of the HCPT@ZIF-8-PDA

To create HCPT@ZIF-8-PDA, 10 mg of HCPT@ZIF-8 was dispersed in a mixture of ethanol and water and stirred for 10 min. Next, Tris-HCl solution (25 mM, 10 mL) was added dropwise to the above solution, followed by 10 mg of DA. The mixture was stirred by a magnetic stirrer for 12 h and then centrifuged (1000 g/rcf) to remove excess DA. The remaining product was washed three times with water to obtain HCPT@ZIF-8-PDA.

### 2.5 Preparation of HCPT@ZIF-8-PDA-FA/FITC

10 mg HCPT@ZIF-8-PDA was evenly dispersed in Tris-HCl solution (25 mM, 10 mL) with 20 mg of FA, and the mixture was stirred overnight for 24 h. After this, 10 mg of FITC was added, and the stirring continued for 48 h. The resulting product was centrifuged and washed three times with deionized water to eliminate any unloaded FITC. The final dispersion was suspended in deionized water to obtain HCPT@ZIF-8-PDA-FA/FITC for further characterization and experiments.

### 2.6 Scanning Electron Microscopy (SEM) Characterization of CPT Nanoparticles

For SEM characterization, the prepared ZIF-8, HCPT@ZIF-8, HCPT@ZIF-8-PDA, and HCPT@ZIF-8-PDA-FA/FITC dispersions were sonicated to ensure even dispersion for 20 min (100 W). The dispersed solutions were then deposited onto clean silicon wafers and allowed to dry adequately. The surface morphology of ZIF-8, HCPT@ZIF-8, HCPT@ZIF-8-PDA, and HCPT@ZIF-8-PDA-FA/FITC was examined using SEM (Merlin Compact, Carl Zeiss, Oberkochen, Germany). In brief, put the sample onto the specimen stage, insert the stage into the SEM sample chamber, and activate the vacuum system to evacuate the chamber to a pressure of from 10^−3^ to 10^−6^ Pa. Align the electron gun, condenser lens, and objective lens. Set the accelerating voltage to 10 kV and adjust the contrast/brightness. Use slow scan speed (>1 min per frame) to enhance the signal-to-noise ratio, and process the images using Zeiss ZEN software (xT V23.2.0, Carl Zeiss, Brno, Czech Republic).

### 2.7 Fourier Transform Infrared Spectrum (FTIR) Characterization of CPT Nanoparticles

For FTIR characterization, 5 mg of ZIF-8, HCPT, HCPT@ZIF-8, HCPT@ZIF-8-PDA, and HCPT@ZIF-8-PDA-FA/FITC were mixed with freeze-dried 495 mg potassium bromide powder (KBr) (W:W = 1:99) and ground evenly. The basic chemical structures of ZIF-8, HCPT, HCPT@ZIF-8, HCPT@ZIF-8-PDA, and HCPT@ZIF-8-PDA-FA/FITC were examined using Fourier transform infrared spectroscopy (Nicolet 5700, Thermo Electron Corporation, Waltham, MA, USA). The wavenumber range was from 4000–400 cm^−1^.

### 2.8 In Vitro Drug Release from HCPT@ZIF-8-PDA-FA/FITC

The *in vitro* drug release behavior of HCPT in HCPT@ZIF-8-PDA-FA/FITC under different pH conditions was investigated using the dialysis bag (Solarbio, Beijing, China) diffusion method. Approximately 4 mg/mL HCPT@ZIF-8-PDA-FA/FITC (20 mg of HCPT@ZIF-8-PDA-FA/FITC was dispersed in 5 mL of phosphate-buffered saline (PBS) was constantly stirred at different pH values (pH = 5.0, 7.4). Samples were collected at specific time intervals, and the released HCPT in the resulting supernatant was measured using UV-visible (TU-1950, Purkinje, Beijing, China) absorbance at 390 nm to calculate the amount of released HCPT.

$$\text{Release}\;\%=\frac{M\;cumulative}{M\;total}\times 100$$

### 2.9 Cytotoxicity Experiment

According to the previously reported method [11], the Water-Soluble Tetrazolium Salt 8 (WST-8) reagent used in the CCK-8 method can be reduced to a highly water-soluble yellow formazan by dehydrogenase present in living cells. However, this reduction reaction does not occur in dead cells. The number of viable cells is directly proportional to the amount of formazan generated by this reduction within a certain range, and the corresponding absorbance value can be measured at 450 nm to reflect the number of viable cells. For the experiment, cells from the MDA-MB-231, BT20, and 4T1 cell lines in the logarithmic growth phase were digested with trypsin (0.25%), and 3000 cells were seeded per well in 96-well plates. The plates were placed in an incubator (5% CO_2_, 37°C) and cultured overnight. Different concentrationsof 9-HCPT (50, 25, 12.5, 078, 0.39, 0 μg/mL) and 9-HCPT nanoparticles (12.5, 0.78, 0.19, 0.02, 0.01, 0 μg/mL) were added by dilution, with three replicate wells set for each concentration. After incubation for 24 h, the cells were irradiated with NIR light (808 nm, 0.4 W/cm^2^) for 3 min, the culture media containing the drugs were removed, and 90 μL of serum-free medium and 10 μL of CCK-8 solution (CK04, Kumamoto, Japan) were added to each well. The plates were returned to the incubator for an additional 2 h. Using a microplate reader (Synergy H1, BioTek, Winooski, VT, USA), the absorbance at 450 nm was measured to evaluate the cell inhibition of the nanoparticles. A growth curve was plotted, and the half-inhibition concentration (IC_50_) was calculated.

### 2.10 Western Blot

In order to measure the protein expression, according to the previously reported method [12], 25 μg protein samples were collected and quantified, then denatured. Electrophoresis was performed, followed by transmembrane and non-fat milk blocking. Incubation with the primary antibody (Rabbit mAb, diluted with 1:1000) and HRP second antibody (anti-rabbit IgG, diluted with 1:3000) was done, and subsequently, the PVDF membrane was probed and washed with Tris-buffered saline with Tween 20 (TBST, 0.1%). The PVDF membrane was then placed into the Bio-Rad gel imager (ChemiDoc MP, Bio-Rad, Hercules, CA, USA). The ECL chemiluminescent solution was applied to cover the entire membrane, and appropriate images were selected after a certain exposure time. Immunoblot density analysis was performed using ImageJ 2.2 software (NIH, Bethesda, MD, USA).

### 2.11 Animal Experiment

30 female mice (BALB/c, 5–6 weeks old, 18–20 g) were provided by Guangdong Zhiyuan Technology Company (SYXK(Yue)2021-0251, Guangzhou, China). All animals were fed sterile food and water in sterile animal feeding facilities with a temperature of 20°C–25°C and a relative humidity of 50%–65%. All animal experiment procedures were approved by the Animal Ethics Committee of Guangdong Medical University (GDY2502311). 4T1 cells were first suspended in serum-free medium, and 0.1 mL of the cell suspension (approximately 1 × 10^7^ cells) was then injected into the left upper axilla of each mouse to establish xenografts. The size of the subcutaneous tumor in each nude mouse was measured with digital Vernier calipers. Nude mice were numbered and randomly divided into five groups: ZIF-8 (Control) group, HCPT@ZIF-8 group, HCPT@ZIF-8-PDA group, HCPT@ZIF-8-PDA-FA/FITC group and HCPT group. In this study, mice were administered drugs every 3 days for 12 consecutive days. Before each administration, the longest (A) and shortest (B) diameters of the subcutaneous tumors in the mice were measured with digital calipers, and the tumor volume (V) was calculated by the equation: V = 0.5 × A × B^2^. After 12 consecutive days of administration, all nude mice were killed by intraperitoneal injection of chloral hydrate and were necropsied on ice packs, and the subcutaneous xenografts were harvested. Each xenograft was weighed on an electronic scale, and the relevant data were recorded. At the same time, part of the tumor tissue was removed and frozen in liquid nitrogen for further experiments.

### 2.12 Statistical Analysis

Statistical analysis was conducted using SPSS 29.0 software (IBM Corp., Armonk, NY, USA). One-way analysis of variance (ANOVA) was used to analyze statistical differences between multiple groups. Each experiment was repeated at least three times, and the results were expressed as mean ± standard error of the mean (SD). Statistical significance was considered at *p*-values less than 0.05.

## 3 Results

### 3.1 Nanoparticle Construction

**Figure 1:**
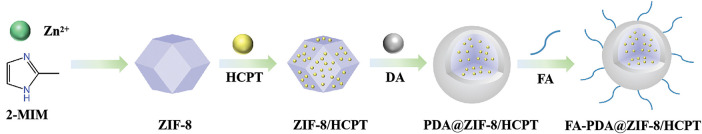
Construction of HCPT@ZIF-8-PDA-FA/FITC nanoparticles. Zeolite imidazole ester backbone material (ZIF-8) was synthesized by stirring at room temperature, yielding ZIF-8 nanoparticles. The ZIF-8 nanoparticles were loaded with HCPT and dispersed in the HCPT methanol solution (HCPT@ZIF-8). Dopamine (DA) and folic acid (FA) were utilized to form the polymer nanoparticles

ZIF-8 was successfully synthesized by stirring at room temperature. The absorbance of HCPT at 390 nm was measured using a UV spectrophotometer to determine the concentration of unloaded HCPT in the centrifugal supernatant. The calculated encapsulation rate was 89.9%, and the DLC was 54.15%. HCPT@ZIF-8 was then dispersed in a mixture of ethanol and water with Tris-HCl solution and dopamine to form HCPT@ZIF-8-PDA. Next, HCPT@ZIF-8-PDA was mixed with folic acid and stirred overnight, then FITC was added, followed by additional stirring and centrifugation. After washing with deionized water three times to remove the unloaded FITC, HCPT@ZIF-8-PDA-FA/FITC nanoparticles were obtained (Fig. 1).

### 3.2 Scanning Electron Microscopy Characterization of Nanoparticles

**Figure 2:**
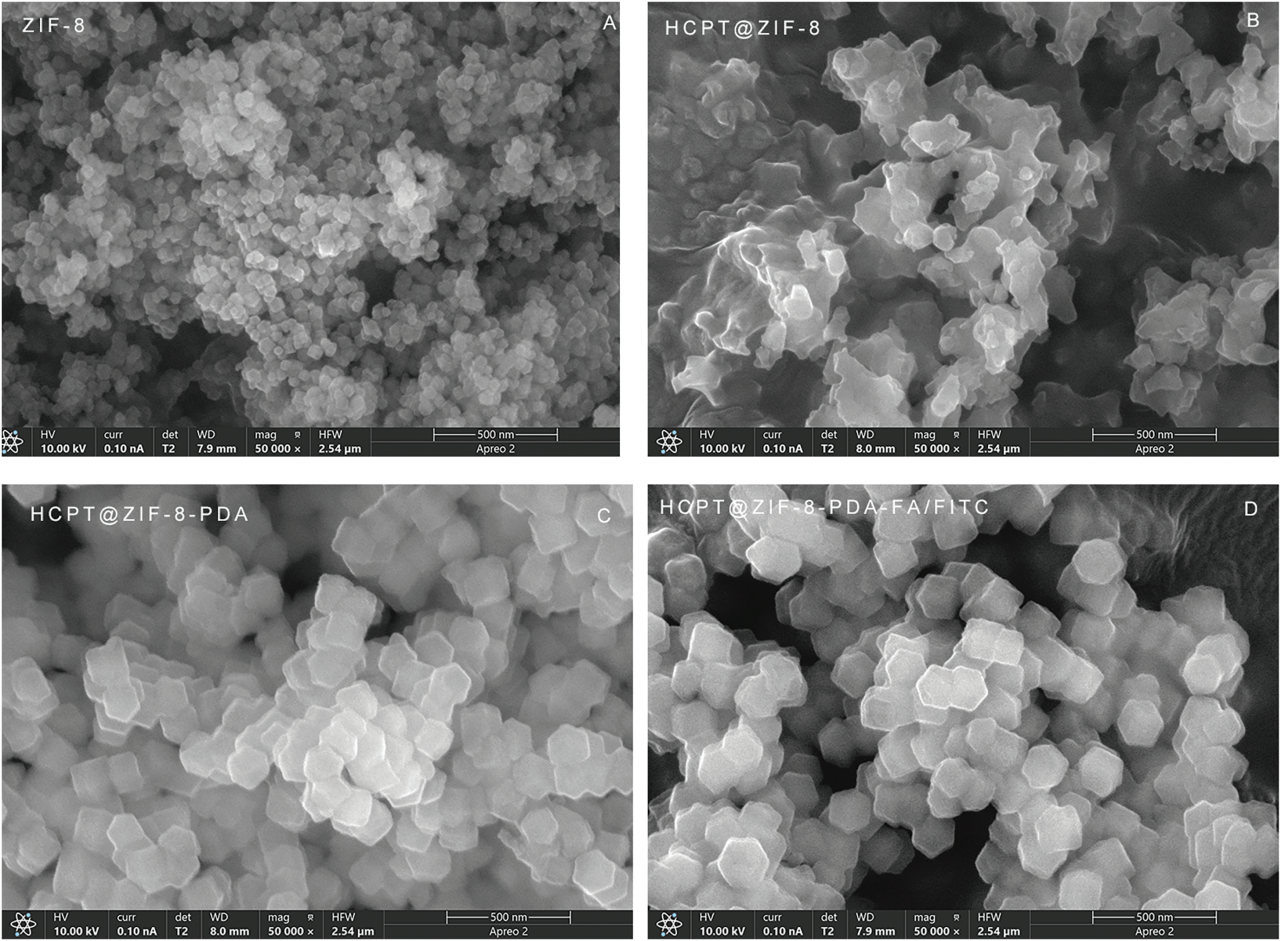
Scanning electron microscopy (SEM) characterization of the nanoparticles. (**A**) Crystal shape of ZIF-8; (**B**) Crystal structure of HCPT@ZIF-8; (**C**) Crystal structure of HCPT@ZIF-8-PDA; (**D**) Crystal structure of HCPT@ZIF-8-PDA-FA/FITC

As shown in Fig. 2, the SEM results demonstrated that the synthesized ZIF-8 crystals exhibited a regular shape with a rhombic dodecahedral structure and a uniform particle size distribution, mainly concentrated at 60 nm. The size of ZIF-8 nanoparticles was approximately 85 nm (Fig. 2A). Importantly, HCPT@ZIF-8 retained the polyhedral structure of ZIF-8 after loading with HCPT (Fig. 2B). Upon self-polymerization of dopamine onto HCPT@ZIF-8, the size of HCPT@ZIF-8-PDA increased to 120 nm (Fig. 2C), and the surface became less smooth, displaying a slight degree of collapse and folding. Following the connection with the target molecule FA and the fluorescent molecule FITC, the size of HCPT@ZIF-8-PDA-FA/FITC nanoparticles remained relatively unchanged, while the surface appeared significantly more collapsed (Fig. 2D).

### 3.3 Infrared Detection Results of Nanoparticles

**Figure 3:**
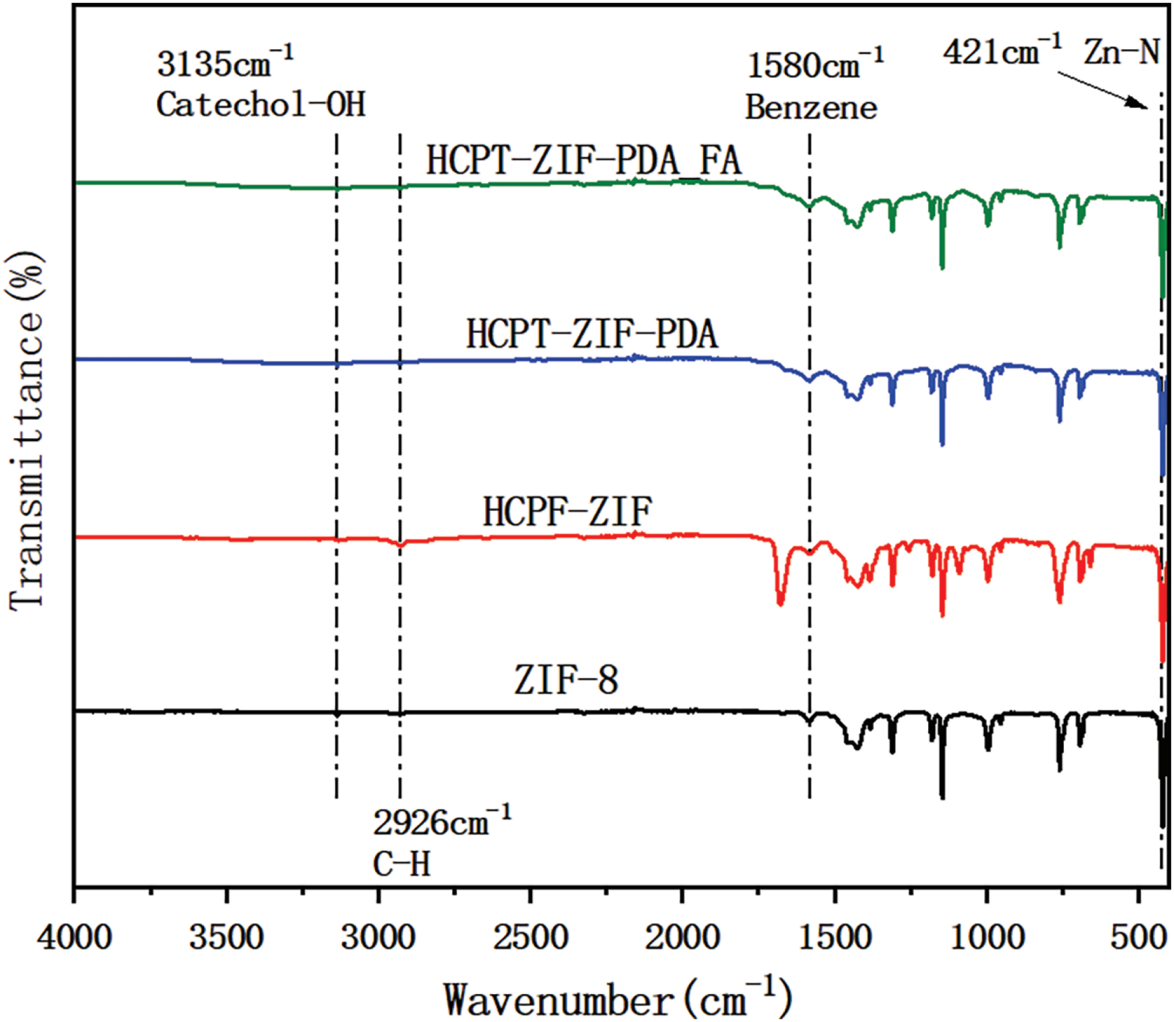
Infrared detection results of the nanoparticles

FTIR analysis was conducted to validate the successful synthesis of the nanoparticles and investigate their structural and chemical bonds. The spectra of ZIF-8, HCPT@ZIF-8, HCPT@ZIF-8-PDA, and HCPT@ZIF-8-PDA-FA/FITC were analyzed. The ZIF-8 spectrum showed characteristic peaks at 421 cm^−1^, corresponding to the Zn-N group formation, and at 2926 cm^−1^, corresponding to the C-H group in the imidazole. Additionally, a peak at 1580 cm^−1^ was observed, corresponding to the backbone vibration of the benzene ring in the HCPT structure. After loading with PDA, a characteristic peak attributed to PDA catechol-OH appeared at 3135 cm^−1^. These results confirmed the successful synthesis of ZIF-8 and the subsequent PDA and FA/FITC loading onto the nanoparticles (Fig. 3).

### 3.4 In Vitro Drug Release Results of Nanoparticles

**Figure 4:**
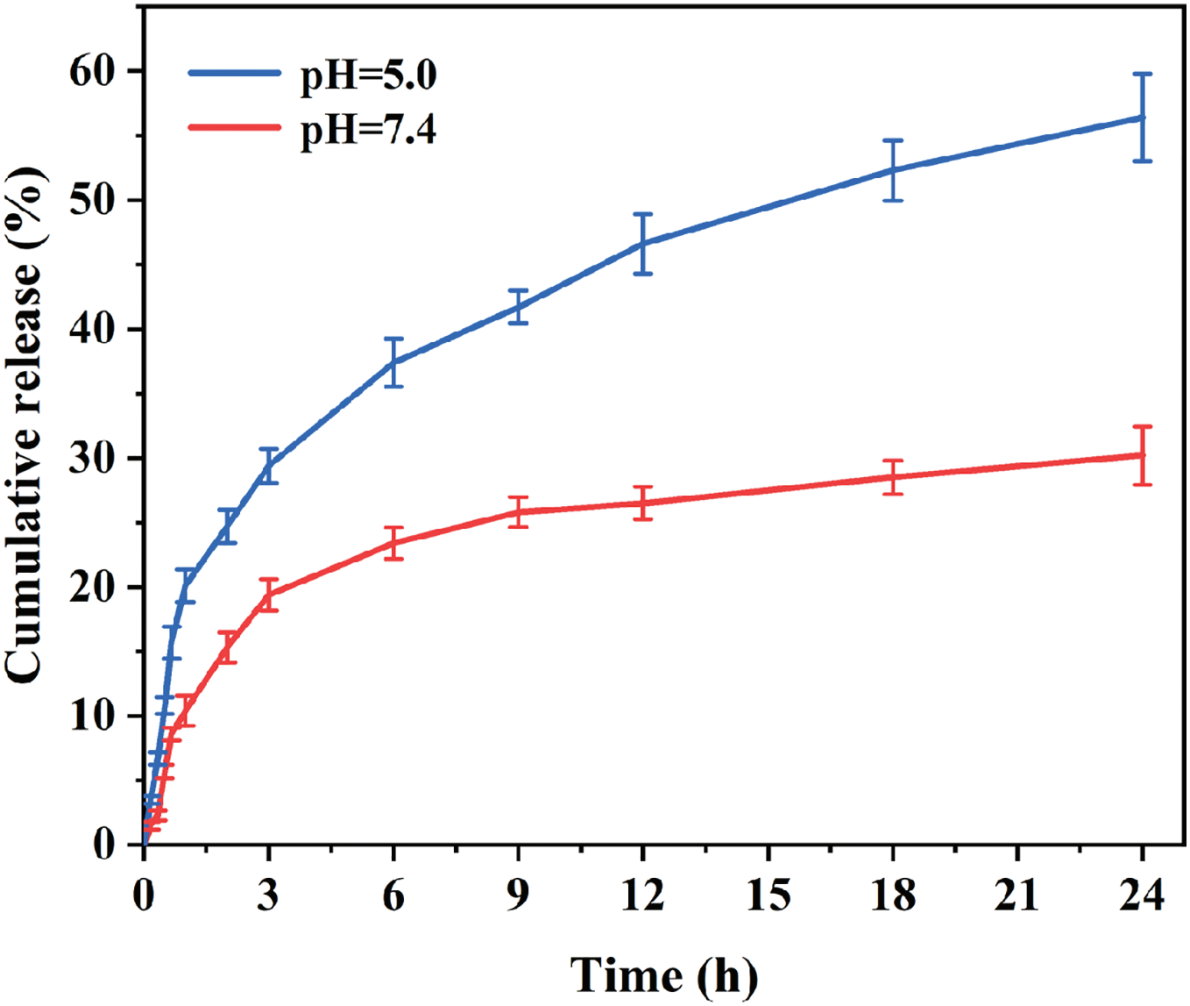
*In vitro* drug release of the nanoparticles

The drug release capacity of HCPT@ZIF-8-PDA-FA/FITC was assessed at different pH values. The drug release curves within 24 h were tested, and the results are shown in Fig. 4. Over time, drug release increased in a time-dependent manner. In an acidic environment, HCPT was continuously released from HCPT@ZIF-8-PDA-FA/FITC, reaching a cumulative release of 56.40% ± 3.37% after 24 h, which was significantly higher than observed in a neutral environment (30.20% ± 2.27%, *p* = 0.004). The observed pH-dependent drug release behavior can be attributed to the structural stability of ZIF-8 in a neutral physiological environment. However, in an acidic environment, the protonation of the 2-methylimidazole connecting chain in ZIF-8 disrupts the coordination interaction between the zinc ion and the imidazole ring, leading to the gradual degradation of the ZIF-8 structure and subsequent drug release.

### 3.5 HCPT@ZIF-8-PDA-FA/FITC Reduced Cytotoxicity and Inhibited Tumor Proliferation

**Figure 5:**
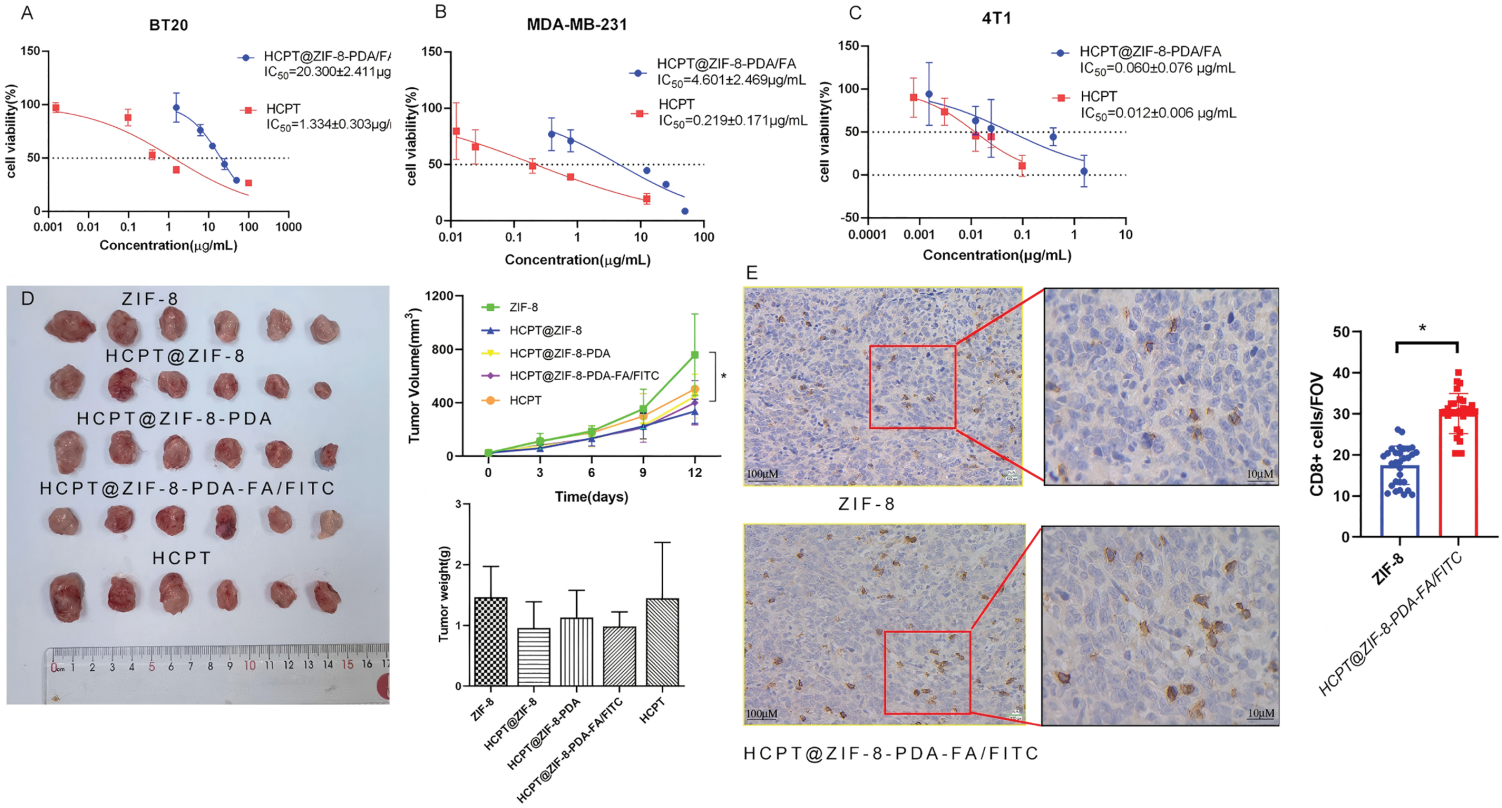
HCPT-ZIF-8-PDA-FA/FITC showed cytotoxicity *in vitro* and *in vivo*. (**A**) The IC_50_ values of CPT nanoparticles were 20.3 μg/mL for BT20 cells; (**B**) The IC_50_ values of CPT nanoparticles were 4.6 μg/mL for MDA-MB-231 cells; (**C**) the IC_50_ value was 0.06 μg/mL for 4T1 cells; (**D**) HCPT-ZIF-8-PDA-FA/FITC could inhibit tumor proliferation *in vitro* (N = 6, vs. ZIF-8: control; *p < 0.05); (**E**) HCPT@ZIF-8-PDA-FA/FITC induce CD8^+^ T cell infiltrates compare to ZIF-8 group (N = 30, vs. ZIF-8: control; *p < 0.05)

To address the issue of CPT’s high toxicity and its limitations in application, we compared the cytotoxicity of CPT nanoparticles and free CPT on breast cancer cells. *In vitro* cytotoxicity was assessed using the CCK-8 assay. Breast cancer cells (BT20, MDA-MB-231, and 4T1) were seeded in 96-well plates at a density of 3000 cells/well. Different concentrations (0, 20, 40, 80, and 100 μg/mL) of HCPT@ZIF-8-PDA-FA/FITC were added to the wells and incubated in the incubator for 24 h. Subsequently, the cells were irradiated with NIR light (808 nm, 0.4 W/cm^2^) for 3 min. After further incubation with CCK-8 solution (10 μL) for 2 h, the absorbance values at 450 nm were measured using a microplate reader. The IC_50_ values of HCPT nanoparticles (HCPT@ZIF-8-PDA/FA) were 20.300 ± 2.411 μg/mL and 4.601 ± 2.469 μg/mL for BT20 (Fig. 5A) and MDA-MB-231 (Fig. 5B), respectively, while the IC_50_ value was 0.060 ± 0.076 μg/mL for the mouse breast cancer cell line 4T1 (Fig. 5C). In contrast, the IC_50_ values of free HCPT for human breast cancer cell lines BT20 and MDA-MB-231 were 1.334 ± 0.303 μg/mL and 0.219 ± 0.171 μg/mL, respectively, and the corresponding value for the 4T1 cell line was 0.012 ± 0.006 μg/mL. Then, we evaluated the anti-cancer effect of HCPT@ZIF-8-PDA-FA/FITC *in vivo*. As shown in Fig. 5D, nude mice were numbered and randomly divided into five groups: ZIF-8 (Control) group, HCPT@ZIF-8 group, HCPT@ZIF-8-PDA group, HCPT@ZIF-8-PDA-FA/FITC group and HCPT group. After 12 consecutive days of subcutaneous administration of drugs, the body weight in treated mice was not significantly different from that of those mice in the control group (ZIF-8), while the tumor volumes in HCPT@ZIF-8-PDA-FA/FITC treated mice were significantly lower than those mice in the control (ZIF-8) group (*p* = 0.0217). We also found immune infiltrates in the HCPT@ZIF-8-PDA-FA/FITC group in Fig. 5E (*p* = 0.002). These results demonstrate that using zeolite imidazole ester backbone material for constructing CPT nanoparticles could significantly reduce the cytotoxicity of CPT and inhibit tumor proliferation.

### 3.6 Nanoparticles Activate the cGAS/STING Signaling Pathway

**Figure 6:**
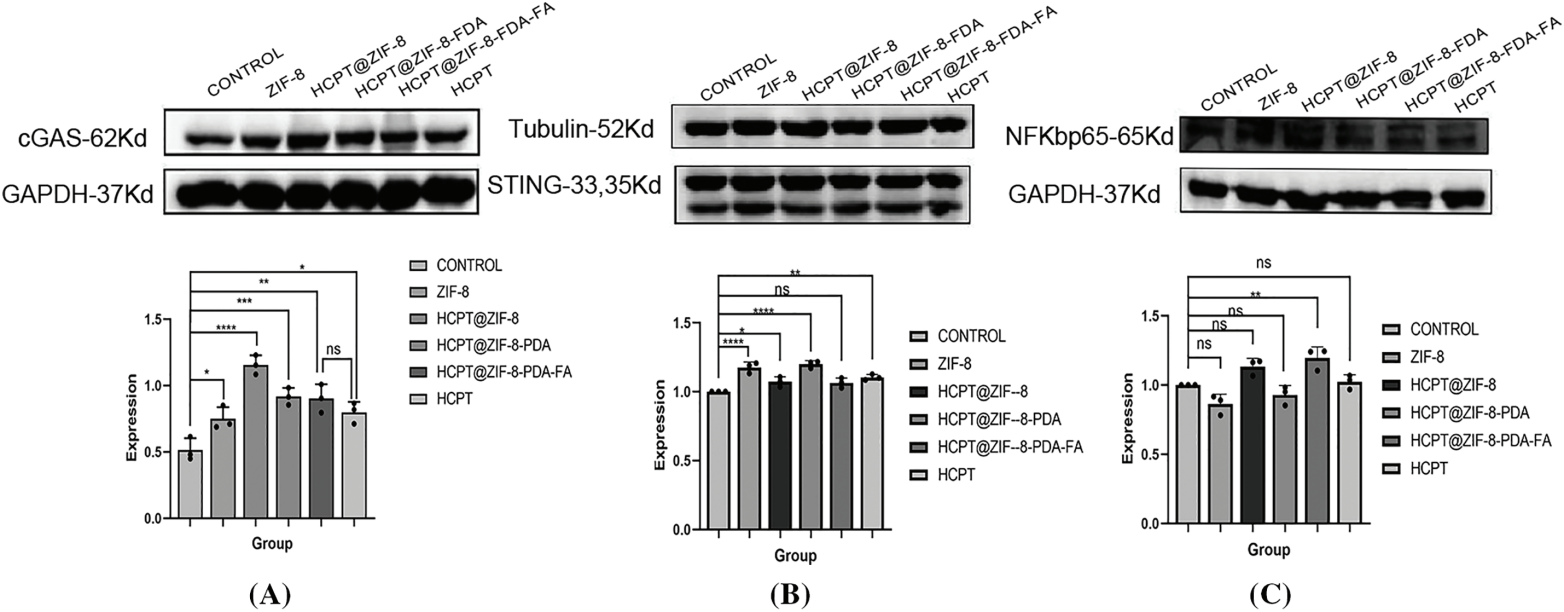
Activation of the cGAS/STING signaling by HCPT-ZIF-8-FDA-FA. (**A**) Protein expression of cGAS; (**B**) Protein expression of STING; (**C**) Protein expression of NF-κB-p65 (vs. ZIF-8: control; ns, no significant; *p < 0.05, **p < 0.01, ***p < 0.001, ****p < 0.0001)

The cGAS/STING signaling pathway plays a crucial role in tumor immunotherapy [13]. It involves the cGAS protein, which is responsible for synthesizing the second messenger cyclic GMP-AMP (cGAMP), and the STING protein, which acts as the direct receptor of cGAMP [14]. Activation of the cGAS/STING pathway in tumor cells leads to the secretion of various aging-related secretory phenotypes, such as proinflammatory cytokines, chemokines, growth factors, and proteases, ultimately inducing cellular senescence and inhibiting tumor proliferation [15]. Given the importance of improving drug bioavailability and reducing side effects, various drug delivery systems have been designed, and polymer nanocarriers have emerged as promising candidates due to their easy surface modification and synthesis, enhancing the pharmacological activity of compounds [16]. This study evaluated the expression of cGAS, STING, and NF-κB-p65 by nanoparticles in human breast cancer MDA-MB-231 cells. Finally, we observed that, compared to free CPT, the nanoparticles upregulated cGAS expression, suggesting that the nanoparticles may activate the cGAS/STING signaling pathway in tumor cells, inhibiting tumor cell invasion (Fig. 6).

## 4 Discussion

CPT, an alkaloid isolated from *Camptotheca acuminata* in 1966, has demonstrated clinical efficacy against various cancers, such as gastric, liver, bladder, colon, ovarian, leukemia, and chorionic epithelial cancers [17]. However, the lactone ring in CPT’s structure is susceptible to rapid hydrolysis into the inactive open-ring form under physiological conditions, limiting its efficacy in clinical tumor treatments [18]. Additionally, CPT’s poor water solubility and susceptibility to light-induced decomposition restrict its clinical application [19]. Despite its potent anticancer activity, CPT and its current dosage forms suffer from toxic side effects, including poor cell selectivity, myelosuppression, gastrointestinal toxicity, and hemorrhagic cystitis [20].

The drug delivery system plays a crucial role in enhancing the efficacy of anti-tumor drugs by increasing drug solubility, enabling controlled release, improving stability, and enhancing tumor targeting [21,22]. Compared to traditional preparations, drug delivery systems can significantly enhance the bioavailability of insoluble drugs, increase drug concentration at the tumor site, and reduce the toxic side effects of drugs [23]. For CPT drugs, their instability under physiological conditions and the pH-dependent nature of their essential lactone ring pose significant challenges in achieving effective antitumor activity [24]. Protecting the lactone structure of CPT and preventing its hydrolytic inactivation are key aspects to improve its efficacy. One approach is encapsulating CPT in liposomes, which increases its inner ester ring stability and maintains its active structure. However, conventional liposomes are easily cleared by the reticuloendothelial system and lack efficient targeting capabilities [25]. CPT-SS-GPCs liposomes have been synthesized and assembled using disulfide bonding to address these issues. These liposomes effectively release the drug in the reductive internal environment of tumor cells [26]. *In vitro* and *in vivo* evaluations have shown that CPT-SS-GPCs liposomes significantly enhance the drug load of CPT, exhibit high *in vivo* stability, and enable rapid release within tumor cells [27,28]. This approach has shown promise in tumor treatment, although challenges related to phospholipid oxidation, controlled release, and poor stability must be addressed to achieve its potential fully.

Despite various attempts to overcome the limitations of CPT drugs by developing different delivery systems like liposomes, polymer micelles, nanoparticles, microspheres, and high molecule precursor drugs, several challenges remain to be addressed, encompassing low drug loading, poor stability, limited control over drug release, and specific toxicities associated with nanocarriers (e.g., gastrointestinal tract irritation, neutropenia), as well as tumor multi-drug resistance [29,30]. This study addressed these challenges by synthesizing zeolite imidazole ester backbone material through a simple room-temperature stirring process, resulting in ZIF-8 nanoparticles. We further dispersed ZIF-8 nanoparticles in an HCPT methanol solution to obtain HCPT-loaded ZIF-8 nanoparticles (HCPT@ZIF-8). The encapsulation rate and drug loading of the ZIF-8 nanoparticles were 89.9% and 54.15%, respectively. To improve drug delivery efficiency and reduce side effects on normal tissues, we incorporated dopamine and folic acid into the polymer nanoparticles, allowing for a distinct release mode under either pH conditions or near-infrared laser stimulation. Accordingly, drug nanoparticles could be quickly released at the tumor site, achieving higher drug concentrations and enhancing the therapeutic effect. Through experimental validation, we observed that HCPT@ZIF-8-PDA-FA/FITC significantly reduced cytotoxicity compared to traditional HCP T delivery. Meanwhile, we observed upregulation of cGAS expression in tumor cells, indicating effective uptake and targeted drug delivery of HCPT@ZIF-8-PDA-FA/FITC nanoparticles. This promising approach allows for a synergistic therapy involving photothermal and chemotherapy. This study’s findings substantiated that the HCPT@ZIF-8-PDA-FA/FITC nanoparticles, created by integrating prodrug strategy and nanotechnology with chemotherapy and immunotherapy, exhibited enhanced effectiveness while minimizing cytotoxicity to normal tissues. These nanoparticles also contributed to improving the tumor microenvironment, enhancing the immune response, and enabling the combination treatment with combined immunodetection point inhibitors for tumors [31,32].

In this study, HCPT@ZIF-8-PDA-FA/FITC nanoparticles have shown some efficacy in inhibiting breast cancer growth. However, there are still some limitations. Such as high interstitial fluid pressure, dense extracellular matrix (ECM), and acidic pH hinder nanoparticle penetration into deep tumor regions. Poorly understood biodistribution and slow clearance of non-degradable HCPT@ZIF-8-PDA-FA/FITC nanoparticles raise concerns about organ accumulation and chronic toxicity. Further investigations are essential to understand the pharmacokinetic behavior of these nanoparticles in living organisms. Determining the optimal dose ratio and timing of chemotherapeutic drugs and immune checkpoint inhibitors remains crucial for maximizing their therapeutic potential.

## Data Availability

All data generated or analyzed during this study are included in this published article.
